# Supplementary material for: A Seroepidemiological Survey of Corynebacterium pseudotuberculosis Infection in South Tyrol, Italy
Source: Pathogens. 2022 Nov 9;11(11):1314. doi: 10.3390/pathogens11111314 (PMC9696596; doi:10.3390/pathogens11111314)
Supplement: Supplementary file 1 [file pathogens-11-01314-s001.zip › pathogens-1942624-supplementary.pdf]

Table S1. Working correlation value of the multivariable GEE model.

Model	Correlation/ Covariance		Estimate
**Marginal model**	**Exchangeable Working Correlation**	ρ	0.2374

Table S2. Type III Tests: significant variables of the multivariable GEE model.

		Type III Tests	
**Variables**	**Num DF**	Chi-Square	Pr > ChiSq
Race	3	3.63	0.3041
Age (year)	1	24.27	<.0001
Age*Race	3	17.20	0.0006

Table S3. Parameter estimates of the multivariable GEE model.

Analysis Of marginal model (GEE): Parameter Estimates							
**Parameter**		**Estimate**	**Standard Error**	**95% Confidence Limits**		**Z**	**Pr > |Z|**
Intercept		-1.7726	0.3956	-2.5479	-0.9973	-4.48	<.0001
Race	Chamois Coloured goat	0.0847	0.5380	-0.9697	1.1391	0.16	0.8749
Race	Crossbreed	-0.0755	0.4280	-0.9144	0.7634	-0.18	0.8600
Race	Passirian goat	-0.5079	0.4079	-1.3074	0.2916	-1.25	0.2131
Race	Saanen	0	.	.	.	.	.
age_year		0.1194	0.0732	-0.0240	0.2628	1.63	0.1027
age_year*Race	Chamois Coloured goat	0.0772	0.1101	-0.1386	0.2931	0.70	0.4830
age_year*Race	Crossbreed	-0.0732	0.0856	-0.2410	0.0946	-0.86	0.3923
age_year*Race	Passirian goat	0.1250	0.0756	-0.0231	0.2731	1.65	0.0981
age_year*Race	Saanen	0	.	.	.	.	.

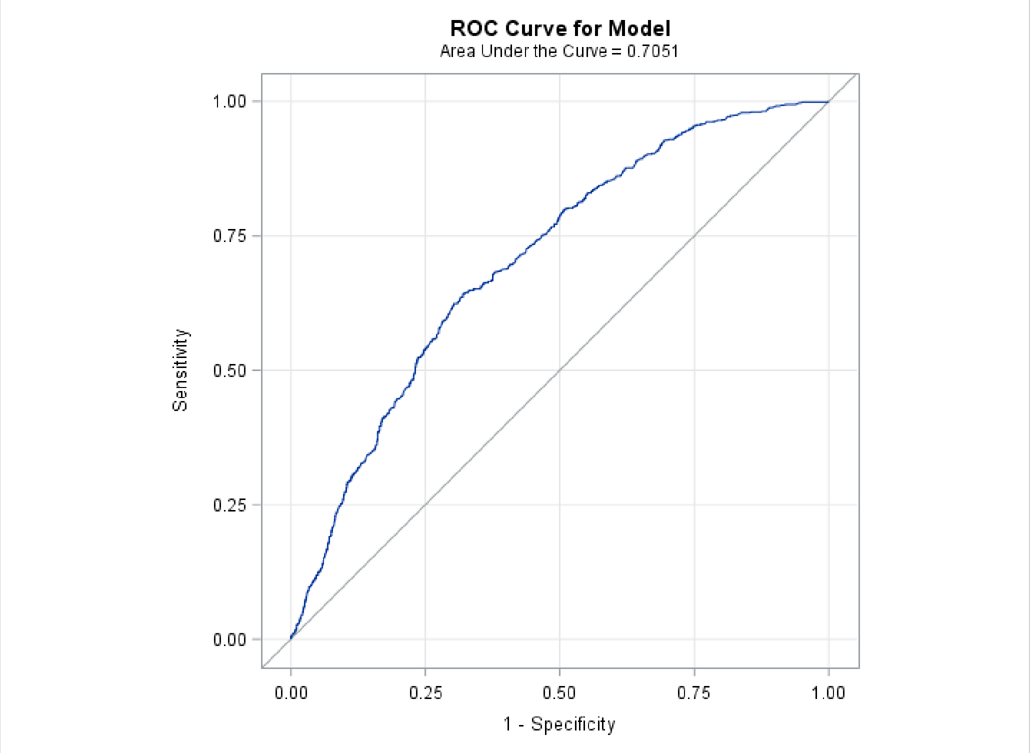

Figure S1. AUC of the ROC curve to evaluate the goodness of the GEE model.
